# Image Object Recognition via Deep Feature-Based Adaptive Joint Sparse Representation

**DOI:** 10.1155/2019/8258275

**Published:** 2019-11-21

**Authors:** Wang Wei, Tang Can, Wang Xin, Luo Yanhong, Hu Yongle, Li Ji

**Affiliations:** ^1^School of Computer and Communication Engineering, Changsha University of Science & Technology, Changsha 410114, China; ^2^Hunan Children's Hospital, Changsha 410000, China; ^3^College of Automotive and Mechanical Engineering, Changsha University of Science & Technology, Changsha 410114, China

## Abstract

An image object recognition approach based on deep features and adaptive weighted joint sparse representation (D-AJSR) is proposed in this paper. D-AJSR is a data-lightweight classification framework, which can classify and recognize objects well with few training samples. In D-AJSR, the convolutional neural network (CNN) is used to extract the deep features of the training samples and test samples. Then, we use the adaptive weighted joint sparse representation to identify the objects, in which the eigenvectors are reconstructed by calculating the contribution weights of each eigenvector. Aiming at the high-dimensional problem of deep features, we use the principal component analysis (PCA) method to reduce the dimensions. Lastly, combined with the joint sparse model, the public features and private features of images are extracted from the training sample feature set so as to construct the joint feature dictionary. Based on the joint feature dictionary, sparse representation-based classifier (SRC) is used to recognize the objects. Experiments on face images and remote sensing images show that D-AJSR is superior to the traditional SRC method and some other advanced methods.

## 1. Introduction

Sparse representation has its unique advantages in signal processing, image processing, computer vision, pattern recognition, and so on. Image recognition based on sparse representation can be divided into two parts: sparse representation and classification recognition. First, we need to build a dictionary to represent the test samples, and then use the sparse representation coefficient and classification dictionary to identify the objects. Wright et al., for the first time, put forward a sparse representation-based classifier (SRC) [1]. SRC used the original training samples as dictionaries to represent the test samples linearly and then calculated the sparse representation coefficients of the test samples. It used sparse representation coefficients and training samples to calculate all kinds of reconstruction residuals, so the test samples can be identified according to the minimum reconstruction residuals.

Lai et al. proposed a tensor feature extraction method based on multilinear sparse principal component analysis (MSPCA). The key operation of MSPCA is to rewrite the multilinear PCA (MPCA) into multilinear regression forms and relax it for sparse regression. Moreover, it inherits the sparsity from the sparse principal component analysis (SPCA), and it can iteratively learn a series of sparse projections, achieving good results in face recognition [2]. By introducing the sparsity or *l*_1_-norm learning, Lai et al. proposed a unified sparse learning framework, which further extends the locally linear embedding-based methods to sparse cases. This method achieves good results in image recognition, especially in the case of small samples [3]. There is also a generalized robust regression (GRR) method for jointly sparse subspace learning. By incorporating the elastic factor on the loss function, GRR can enhance the robustness to obtain more projections for feature selection or classification and have better robustness in face recognition [4].

Convolutional neural network (CNN) is a machine learning model under deep supervised learning. For image recognition, CNN can directly use the image data as input data without manual preprocessing and additional feature extraction. Therefore, CNN has achieved good recognition effects. CNN is very suitable for extracting image features as it can extract a variety of image features including texture, shape, color, and image topology.

Jiajia et al. proposed a new CNN-GRNN model for image classification and recognition. This model used a simple CNN model for image feature extraction, and then used a general regression neural network (GRNN) model for classification [5]. Lu and Linghua proposed a face recognition method based on discriminant dictionary learning, which used a Gabor filter to learn the new dictionary and classified the images with sparse representation [6]. Mahoor et al. proposed a face motion combination recognition framework based on sparse representation and used the average Gabor feature of motion combination to establish an ultracomplete dictionary to improve the recognition accuracy of various actions [7].

To some extent, the aforementioned researches have improved the recognition efficiency, but they also have their own limitations. For example, only using the CNN model for image recognition will both take a lot of time to adjust parameters and require a large number of training samples [8]. Sometimes, it is difficult to obtain a large number of experimental samples that meet the requirements. On the contrary, the sparse representation of the traditional dictionary introduced above mostly used traditional features, which cannot meet the requirement of high recognition rate in many cases. In view of these situations, we improve the traditional dictionary into an extended dictionary and use deep features as the atoms in the dictionary to propose the D-AJSR approach.

D-AJSR is a data-lightweight classification framework with relative high recognition rate. At the same time, compared with artificial intelligence methods, D-AJSR can classify and recognize objects well with few training samples.

## 2. Joint Sparsity Model

### 2.1. Classification Method Based on Sparse Representation

SRC is a classification and recognition framework for face images first proposed by Wright et al. [1], which has been gradually applied to other image classification and recognition. If there are *n* training samples *X*=[*x*_1_, *x*_2_,…, *x*_*n*_](*x*_*i*_ ∈ *R*^*m*^, general *m* << *n*) belong to *k* classes, then the entire training data set can be expressed as

(1) $$\begin{array}{c} W=\left[W_{1},W_{2},\ldots ,W_{k}\right]\in R^{m\times n}, \end{array}$$

where *W*_*i*_=[*v*_*i*,1_, *v*_*i*,2_,…, *v*_*i*,*n*_*i*__], *v*_*i*,*j*_ is the *j*th sample of the *i*th class, and *n*_*i*_ is the sample number of the *i*th class.

Based on the theory of sparse representation (SR), a new test sample *y* ∈ *R*^*m*^ in class *i* can be linearly expressed by the training sample *W*_*i*_ as follows [9]:

(2) $$\begin{array}{c} y=\alpha_{i,1}v_{i,1}+\alpha_{i,2}v_{i,2}+\cdots +\alpha_{i,n_{i}}v_{i,n_{i}}, \end{array}$$

where *α*_*i*,*j*_ ∈ *R* is the sparse representation coefficient of *y*, *j*=1,2,…, *n*_*i*_.

Without considering the noise, formula (2) can be written as

(3) $$\begin{array}{c} y=Wx, \end{array}$$

where *x*=[0,…,0, *α*_*i*,1_, *α*_*i*,2_,…,*α*_*i*,*n*_*i*__, 0,…,0]^*T*^ ∈ *R*^*n*^.

In order to get the sparsest *x*, SRC needs to solve the following *l*_1_ minimization problem:

(4) $$\begin{array}{c} x^{\prime }=\arg\min{\left\|x\right\|}_{1}\\ \text{s.t. }Wx=y, \end{array}$$

where *x*′ is the coefficient vector and ‖*x*‖_1_=∑_*i*_|*x*_*i*_| is *l*_1_ norm.

For each class, we can construct a mapping function *δ*_*i*_(*x*′)=[0,…, 0, *x*_*i*_, 0,…, 0] to represent the nonzero element selected from the coefficient vector *x*′ corresponding to class *i*. The test sample reconstructs the representation with the sparse coefficient as *y*′=*Wδ*_*i*_(*x*′). Then, *y* is classified to class(*i*) by using the minimum residual:

(5) $$\begin{array}{c} \text{class}\left(i\right)=\arg\underset{i}{\min}{\left\|y-W\delta_{i}\left(x^{\prime }\right)\right\|}_{2}, \end{array}$$

where ‖·‖_2_ is *l*_2_ norm and *i*=1,2,…, *k*.

### 2.2. Joint Sparsity Model

Joint sparse model (JSM) was first proposed to encode multiple related signals effectively [10]. In JSM, due to the correlation between the signals, the related signals can be used as one set, and each signal can be represented as a combination of public features and private features on a specific sparse basis. The public feature is the public part of all the signals in one signal set, and the private feature is the characteristic part unique to each signal. So, the *j*th signal can be represented by the public features of a certain set and its own private features:

(6) $$\begin{array}{c} y_{j}=z_{c}+z_{j},\;j\in \left\{1,2,3,\ldots ,J\right\}, \end{array}$$

where *z*_*c*_ represents the public features and *z*_*j*_ represents the private features of the *j*th signal.

Assuming that all images can be divided into *K* classes and there are *J* training images in each category, the *j*th image in the *i*th class can be expressed as *y*_*i*,*j*_. If an image is represented as a one-dimensional column vector, the image in the *i*th class can be represented as *y*_*i*_=[*y*_*i*,1_, *y*_*i*,2_,…,*y*_*i*,*J*_]^*T*^. According to JSM, the *j*th image in the *i*th class can be represented as

(7) $$\begin{array}{c} y_{i,j}=z_{i}^{c}+z_{i,j}^{i}, \end{array}$$

where *z*_*i*_^*c*^ represents the public features of the images in the *i*th class and *z*_*i*,*j*_^*i*^ represents its own private features [11]. If Ψ ∈ *R*^*N*×*N*^ is the orthonormal basis that can sparse represent the training image, then formula (7) can be written as

(8) $$\begin{array}{c} \theta_{i,j}=\Psi z_{i}^{c}+\Psi z_{i,j}^{i}=\theta_{i}^{c}+\theta_{i,j}^{i}, \end{array}$$

where *θ*_*i*,*j*_=Ψ*y*_*i*,*j*_ is the sparse representation of *y*_*i*,*j*_ on transform basis Ψ and *θ*_*i*_^*c*^=Ψ*z*_*i*_^*c*^ and *θ*_*i*,*j*_^*i*^=Ψ*z*_*i*,*j*_^*i*^ represent the sparse representation of the public part and the private part on basis Ψ, respectively. If both sides of formula (8) are left multiplied by Ψ^*T*^, then formula (8) is changed to Ψ^*T*^*θ*_*i*,*j*_=Ψ^*T*^Ψ*z*_*i*_^*c*^+Ψ^*T*^Ψ*z*_*i*,*j*_^*i*^=Ψ^*T*^*θ*_*i*_^*c*^+Ψ^*T*^*θ*_*i*,*j*_^*i*^=*z*_*i*_^*c*^+*z*_*i*,*j*_^*i*^. Combined with formula (7), *y*_*i*,*j*_=Ψ^*T*^*θ*_*i*_^*c*^+Ψ^*T*^*θ*_*i*,*j*_^*i*^, so the joint representation of the image can be expressed as

(9) $$\begin{array}{c} \left[\begin{array}{c} y_{i,1}\\ y_{i,2}\\ \vdots \\ \vdots \\ y_{i,J} \end{array}\right]=\left[\begin{array}{ccccc} \Psi^{T} & \Psi^{T} & 0 & \cdots & 0\\ \Psi^{T} & 0 & \Psi^{T} & \cdots & 0\\ \vdots & \vdots & \vdots & \ddots & 0\\ \Psi^{T} & 0 & 0 & \cdots & \Psi^{T} \end{array}\right]\cdot \left[\begin{array}{c} \theta_{i}^{C}\\ \theta_{i,1}^{i}\\ \theta_{i,2}^{i}\\ \vdots \\ \vdots \\ \theta_{i,J}^{i} \end{array}\right]. \end{array}$$

Formula (9) can be simplified as

(10) $$\begin{array}{c} y_{i}=\tilde{\Psi }W_{i}, \end{array}$$

where *y*_*i*_=[*x*_*i*,1_, *x*_*i*,2_,…,*x*_*i*,*j*_]^*T*^ and $W_{i}={\left[\begin{array}{ccccc} \theta_{i}^{c} & \theta_{i,1}^{i} & \theta_{i,2}^{i} & \cdots & \theta_{i,j}^{i} \end{array}\right]}^{T}$. $\tilde{\Psi }=\left[A,B\right]$ is the overcomplete dictionary and consists of two parts: $A={\left[\begin{array}{cccc} \Psi^{T} & \Psi^{T} & \cdots & \Psi^{T} \end{array}\right]}^{T}$ and *B*=diag(*A*). *W*_*i*_ preserves the discriminant information, and its sparse representation can be obtained by solving the (*l*_1_) minimization of the following formula:

(11) $$\begin{array}{c} W_{i}=\arg\min{\left\|W_{i}\right\|}_{1}\\ \text{s.t. }y_{i}=\tilde{\Psi }W_{i}. \end{array}$$

After obtaining *W*_*i*_, the public features of all images in class *i* and the private features of each image can be obtained in the Ψ field according to the inverse transformation:

(12) $$\begin{array}{c} z_{i}^{c}=\Psi^{T}\theta_{{}_{i}}^{c},\\ z_{i,j}^{i}=\Psi^{T}\theta_{i,j}^{i}. \end{array}$$

All public features and all private features form a joint feature dictionary *D*:

(13) $$\begin{array}{c} D=\left[z_{1}^{c},z_{2}^{c},\ldots ,z_{K}^{c},z_{1,1}^{1},\ldots ,z_{1,J}^{1},z_{2,1}^{2},\ldots ,z_{2,J}^{2},\ldots ,z_{K,1}^{k},\ldots ,z_{K,J}^{k}\right]. \end{array}$$

So, we can use the following formula (14) to identify which category the objects belong to:

(14) $$\begin{array}{c} \text{class}\left(i\right)=\arg\underset{i}{\min}{\left\|y-D\delta_{i}\left(x^{\prime }\right)\right\|}_{2}. \end{array}$$

As can be seen from the above, the joint sparse model algorithm only uses two parts to represent each kind of the training image, which effectively reduces the size of storage space.

## 3. Image Object Recognition Based on D-AJSR

**Figure 1:**
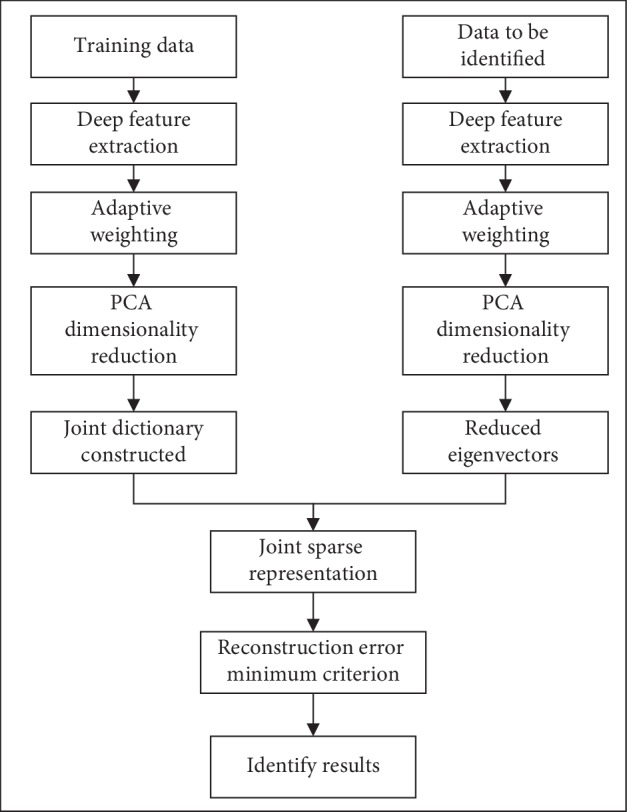
Algorithm framework of D-AJSR.

The algorithm framework of D-AJSR is shown in Figure 1. Unlike JSM summing up private features directly, D-AJSR combines public features and private features into a joint dictionary. Based on this, sparse representation is used to find the sparse solution of the test samples on the adaptive joint dictionary.

### 3.1. Deep Feature Extraction

CNN can automatically extract complex global and local features from images [8]. Therefore, D-AJSR introduces the deep features extracted by CNN into sparse representation to enhance the recognition ability of sparse representation.

**Figure 2:**
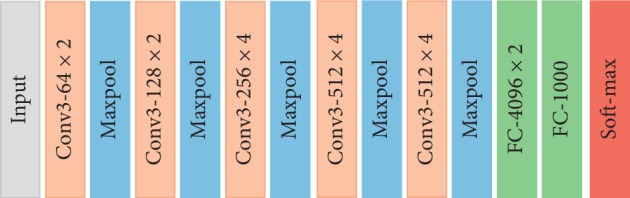
VGG19 network structure.

In this paper, VGG19 is adopted for feature extraction. In the ILSVRC-2014 image classification competition, VGG took the second place with a 7.3% top-5 error rate and the champion of object detection [12]. VGG uses a small convolution kernel of 3 × 3 throughout the construction of the network and superimposes deep networks by superposing 3 × 3 small convolution kernels. The network structure of VGG19 is shown in Figure 2.

**Figure 3:**
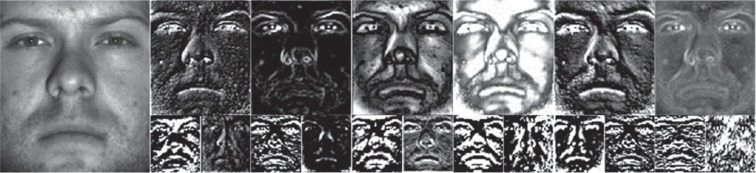
Feature examples extracted by VGG19.

Figure 3 shows the examples of extracted features. The left image is the original image, the upper row shows the features extracted from the first layer, and the lower row shows the features extracted from the second layer. By comparing the extracted features of each layer, it can be found that most textures and detail features are extracted by the shallow network, while the contour and shape features are extracted by the deeper network. Relatively speaking, the deeper the layers are, the more representative the extracted features will be, but the resolution of the feature maps will become lower.

### 3.2. Adaptive Weighted Reconstruction

When constructing the joint dictionary, the object information contained in different samples is also different, and the samples which have larger variance contain more object information. Therefore, we consider increasing the weights of the samples with more object information in the dictionary and reducing the weights of the samples with less object information so as to improve the discrimination ability of the feature dictionary [13].

The feature vector *F*=[*F*_1_, *F*_2_,…,*F*_*n*_]^*T*^ can be transformed as follows after it has been extracted by CNN:

(15) $$\begin{array}{c} {\text{f}}^{\prime }={\left[F_{1}^{\prime }=\frac{\left|F_{1}-\bar{F}\right|}{\bar{F}}F_{1},F_{2}^{\prime }=\frac{\left|F_{2}-\bar{F}\right|}{\bar{F}}F_{2},\ldots ,F_{n}^{\prime }=\frac{\left|F_{n}-\bar{F}\right|}{\bar{F}}F_{n}\right]}^{T}, \end{array}$$

where *F*_*i*_ represents the extracted features of the *i*th image, *F*_2_′ represents the weighted image features, and $\bar{F}=\left(F_{1}+F_{2}+\cdots +F\right)/n$ represents the average of the features.

Formula (15) can adaptively carry out weighted reconstruction and normalization for feature vector elements, which can increase the standard deviation or variance of the feature vectors to a certain extent, help deep feature dictionary containing more recognition information, and improve the recognition efficiency.

### 3.3. Main Steps of D-AJSR

The main steps of D-AJSR are as follows:

1. VGG19 network is used to extract the deep features of the training and the test images.
2. The adaptive weighted reconstruction of feature vectors is carried out to improve the ability of distinguishing feature dictionaries, and the principal component analysis (PCA) method is used to reduce the dimensionality of the reconstructed dictionary.
3. The public features of each class in the feature dictionary and the private features of each image are extracted. All the public features form a matrix *Q*, and all the private features form a matrix *H*. So, we can get the final joint dictionary feature *D*=[*Q*, *H*].
4. Sparse representation of test samples is carried out on the joint feature dictionary, and the sparse coefficient *x*′ is obtained. The feature image *y* of the test sample is reconstructed and identified by using formula (14).

## 4. Experiments and Analysis

In order to verify the validity of D-AJSR, we conduct experiments on the face images and remote sensing images, respectively. The computer used in the experiments is configured as Intel Core i5-3210M @2.5 GHz with 4 GB memory. The experimental platform is Matlab R2017a. In deep feature extraction, we can get 64 global deep features in the first layer and 128 deep features in the second layer. All the experimental results in this chapter are the average results of 10 experiments and D-AJSR(8) represents 8 deep feature maps are used.

### 4.1. Face Image Recognition

In this part, experiments are performed on extended YaleB [14] and AR [15] data sets, respectively. SRC [1], CRC [16], RRC [17], low-rank matrix recovery with structural incoherence (LR) [18], extended SRC (ESRC) [19], discriminative low-rank representation method (DLRR) [20], and sparse dictionary decomposition method (SDD) [21] approaches are compared with D-AJSR in the following experiments.

#### 4.1.1. Experiments on Extended YaleB Data Set

**Figure 4:**
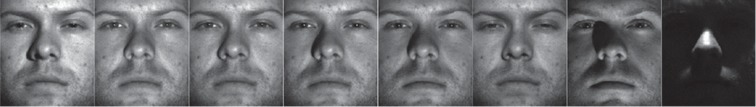
Samples in extended YaleB data set.

Extended YaleB data set contains 2,414 positive images of size 168 × 192 for 38 people under different lighting conditions, part of which are shown in Figure 4. In the experiments, 16 images of each person are randomly selected for training, and the rest are used for testing. The feature dimensions after the PCA process are 25, 50, 75, 100, and 150 respectively. The initial dimension of 8 deep features is 42 × 48 × 8 = 16128. The experiment results are shown in Table 1.

In the experiments, we chose the features obtained from the second layer. In Table 1, bold numbers in each column indicate the highest recognition rate under the same condition. As can be seen from Table 1, D-AJSR maintains a high accuracy in all dimensions, and its performance in 50 dimensions is better than that of other methods in 75, 100, and 150 dimensions. Therefore, D-AJSR can greatly reduce the feature dimension under the same precision requirement.

#### 4.1.2. Experiments on AR Data Set

**Figure 5:**
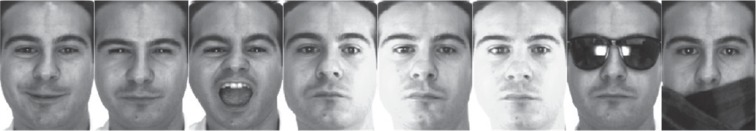
AR data set.

The AR data set contains more than 4000 positive images of 126 people, each with a size of 120 × 165. In the experiments, we use a subset of 2,600 facial images of 100 people, including 50 men and 50 women. Each person has 26 images, which are divided into two separate parts. Each part has 13 pictures, of which 7 are facial expression pictures or unshielded pictures of light changes, 3 are pictures wearing sunglasses, and 3 are pictures camouflaged with scarves, as shown in Figure 5 (the images are selected randomly). In the two parts, we use one part for training and the other part for testing. The feature dimensions of face images are also 25, 50, 75, 100, and 150, respectively. The initial dimension of 8 deep features is 30 × 41 × 8 = 9840.

In the experiments, we chose the features obtained from the second layer. In Table 2, bold numbers in each column indicate the highest recognition rate under the same condition. Because there are samples of wearing sunglasses and camouflaged and the number of these two samples is small, which affects the dictionary training, the recognition rate of our method is lower than that on YaleB data set. As can be seen from Table 2, although the D-AJSR method does not achieve the best effect when the dimensions are 25 and 50, it still remains at a medium level. When the dimensions are 25 and 50, the D-AJSR method does not perform well, mainly because the number of principal components is relatively small and the variance contribution rate is low (less than 0.6). As the representativeness of principal components becomes better, starting from dimension 75, the recognition rate of D-AJSR is better than that of other methods.

In addition, we also compared the experiment results with the locality-constrained and label embedding dictionary learning algorithm (LCLE-DL) [22]. The average recognition rate of LCLE-DL is about 80%, while the average recognition rate of our method is 86.60%. In terms of recognition accuracy, the result of D-AJSR(8) method is relatively better.

### 4.2. Remote Sensing Image Recognition Experiments

**Figure 6:**
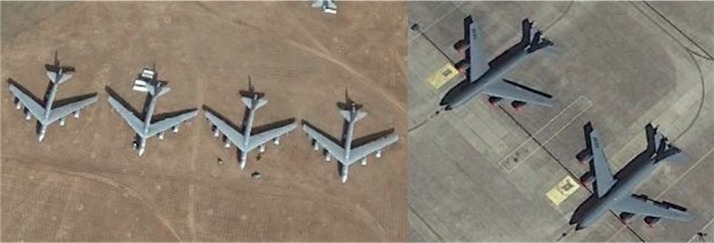
Examples of remote sensing images.

In this part, remote sensing aircraft images are selected from Google Earth 7.1.8 to build data sets for experiments. Remote sensing images of Google Earth are composed of satellite images and aerial images, among which the satellite images come from QuickBird satellite and Landsat-7 satellite, and the aerial images come from BlueSky Company and Sanborn Company and so on. In experiments, images taken at different shooting times and locations are downloaded as data sets.  Figure 6 shows the examples of remote sensing images.

**Figure 7:**
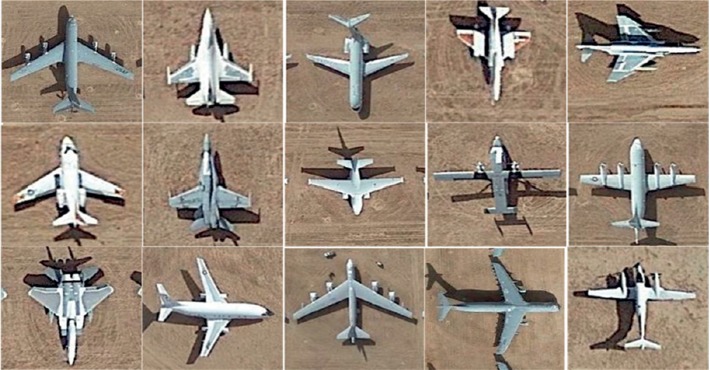
Examples of object images.

The size of a remote sensing image is 170 × 170, and the initial dimension of 64 deep features obtained from the first layer is 85 × 85 × 64 = 462400 (Figure 7). After the PCA process, the feature dimensions of aircraft images are 25, 50, 75, and 100, respectively. In the experiments, the SRC method [1] and the adaptive weighted joint sparse representation classification method (AJRC) [13] are compared with D-AJSR. In these experiments, we choose the features obtained from the first layer. The experimental results are shown in Table 3, and the bold numbers in each column indicate the highest recognition rate under the same condition.

Due to the small number of samples in the data set, the great interference caused by the shadow of the aircraft, and the tire marks on the ground, the recognition rates of all the 3 methods do not reach the better effect as those in the aforementioned experiments. At the same time, because the atoms of the same object are only in 8 directions, the recognition of the object will also be affected. But on the same data set, the effect of D-AJSR is still better than that of the other methods.

### 4.3. Comprehensive Analysis of Experiments

#### 4.3.1. Cumulative Percent of the Principal Components

In the experiments, when using PCA to reduce the dimension of features, the cumulative variance contribution rate of features with different lengths is shown in Table 4. In Table 4, the left column is the number of feature maps selected from the second layer of VGG19, and the results are obtained on YaleB data set.

As can be seen from Table 4, due to different image sizes and different numbers of deep features, the cumulative variance contribution rate of features with the same number is not the same. On the contrary, if the same cumulative variance contribution rate is selected, the feature length will be different. In order to keep the size of the dictionary consistent, we use the same number of principal components in the experiment.

#### 4.3.2. Effect and Efficiency of Different Feature Map Numbers

The recognition rate of D-AJSR varies with the number of depth feature maps. For object recognition on YaleB data set, we use the deep features obtained from the second layer of the VGG19 network, and there are 128 feature maps in total. These feature maps contain different information of the objects. We choose different number of feature maps in experiments, and the recognition results are shown in Table 5. The first row in the table is the number of principal components selected by PCA, and the left column is the number of feature maps selected after deep feature extraction.

The bold numbers in Table 5 are the highest recognition rate in each column. As can be seen from Table 5, when the number of feature maps is small, the recognition rate will increase with the increase of feature maps. When the number of feature maps increases from 1 to 4, the recognition rate increases by 4.89% on average, among which the recognition rate increases by the most when 25 principal components are selected, reaching 8.69%. However, with the increase of feature maps, the improvement of recognition rate is not obvious or even decreases. For example, when the number of deep feature maps increases from 64 to 128, all recognition rates decrease in varying degrees. Because the number of deep feature maps is too large, the original atomic column vectors are too long and the energy is difficult to concentrate, so the resolution of the final feature vector decreases. Therefore, in practical applications, we need to select the appropriate number of deep feature maps.

In addition to the recognition rate of D-AJSR, we also calculate its time efficiency. The experiments are carried out on the remote sensing image data set and compared with SRC and AJRC. The training efficiency results of different approaches on remote sensing images are shown in Table 6, the test efficiency results are shown in Table 7, and the unit of time is second (s). In the experiments, there are 150 training samples and 225 test samples, and the sample size is 170 × 170. The experiments adopt the feature maps of the first layer, and D-AJSR(64) represents 64 deep feature maps are used.

From Tables 6 and 7, it can be seen that the object recognition time of D-AJSR is longer than that of SRC. However, when the number of feature maps is less than 32, the recognition time is shorter than that of AJRC. The feature extraction of 226 deep feature maps in D-AJSR takes about 30 seconds. Considering the results of Tables 5–7, D-AJSR is more advantageous than the other two methods.

From all the aforementioned experiments, we can see that D-AJSR can achieve satisfactory recognition results when the data set is small. Generally speaking, in the recognition of remote sensing objects, VGG and other neural network methods often need thousands of images as training sets for each class, while D-AJSR only need a few images as atoms for each class to output the recognition results. In many cases, it is difficult to obtain a large number of training data for specific tasks, such as the recognition of sensitive objects in special circumstances, identification of unusual objects, and so on. At this time, D-AJSR can give full play to its advantages and provide timely recognition results.

## 5. Conclusions

Aiming at the application requirements of object recognition, we introduce deep features into adaptive joint sparse representation and propose D-AJSR, a data-lightweight classification framework. In order to improve the object recognition rate, the method also adaptively adjusts the atomic weights. Experimental results show that the method has relatively higher recognition rate. On the contrary, since deep feature extraction is more complex than simple change feature extraction, the time consumption of the method will increase correspondingly.

When the number of samples is too small, methods such as deep learning cannot provide reliable identification results due to inadequate training. However, D-AJSR can provide recognition results when there are only a dozen or even a few samples, which provides an effective solution for object recognition without sufficient samples. In addition, after angular rotation expansion of training samples, D-AJSR also has a certain ability of rotating object recognition. In D-AJSR, feature extraction needs some time. Therefore, under the framework of sparse recognition, how to select features that are more expressive and can be extracted quickly is worth our attention in the future.

## Figures and Tables

**Table 1:** Recognition rate (%) on extended YaleB data set.

Dimensions	25	50	75	100	150
LR	75.97	84.39	88.21	89.09	91.14
ESRC	73.86	85.33	88.37	90.20	91.20
CRC	59.64	79.13	84.39	88.82	92.64
SRC	72.98	85.22	88.43	90.48	92.30
RRC	79.40	85.77	90.03	90.81	93.74
DLRR	85.44	89.81	89.92	92.25	93.05
SDD	89.70	92.03	92.41	92.69	92.75
D-AJSR(8)	**93.16**	**96.05**	**96.84**	**96.58**	**97.37**

**Table 2:** Recognition rate (%) on AR data set.

Dimensions	25	50	75	100	150
LR	68.57	84.14	86.00	88.71	88.00
ESRC	63.14	80.43	85.43	86.14	87.29
CRC	56.43	78.86	86.57	88.86	91.29
SRC	64.29	81.29	88.43	89.29	90.29
RRC	69.57	83.14	89.14	90.57	91.43
DLRR	75.71	**88.14**	89.43	91.00	91.86
SDD	**75.86**	87.29	89.71	91.71	93.00
D-AJSR(8)	67.10	86.00	**90.70**	**94.10**	**95.10**

**Table 3:** Recognition rate (%) of remote sensing aircraft images.

Dimensions	25	50	75	100
SRC	62.00	63.56	65.33	66.00
AJRC	70.62	72.00	76.67	78.67
D-AJSR(64)	**71.33**	**75.53**	**77.33**	**80.65**

**Table 4:** Variance contribution (%) of different quantity feature maps on YaleB data set.

Dimensions	25	50	75	100	150
1	57.75	72.97	80.69	85.69	91.92
4	45.60	62.65	71.35	77.28	85.30
8	45.45	63.67	71.85	77.54	85.33
16	42.19	60.13	68.51	74.52	82.95
32	40.56	58.02	66.59	72.76	81.56
64	41.40	58.71	67.14	73.21	81.90
128	41.72	58.79	67.18	73.22	81.90

**Table 5:** Accuracy (%) of different quantity feature maps on YaleB data set.

Dimensions	25	50	75	100	150
1	82.63	90.00	92.11	93.42	93.42
4	91.32	95.26	96.05	96.58	96.84
8	**93.16**	**96.05**	**96.84**	96.58	97.37
16	**93.16**	93.32	93.32	97.11	97.37
32	**93.16**	95.26	96.32	97.11	97.63
64	92.89	**96.05**	96.58	**97.63**	**97.89**
128	90.79	94.21	94.47	95.00	95.26

**Table 6:** Training efficiency (s) of different approaches on remote sensing images.

Dimensions	25	50	75	100
SRC	1.2649	1.2901	1.2758	1.2833
AJRC	49.734	58.775	78.598	115.08
D-AJSR(1)	22.437	33.191	50.976	93.049
D-AJSR(4)	24.301	33.721	55.423	95.125
D-AJSR(8)	27.216	37.335	59.520	97.495
D-AJSR(16)	32.846	43.249	63.786	103.30
D-AJSR(32)	43.855	55.201	76.756	118.29
D-AJSR(64)	63.104	72.078	94.864	128.94

**Table 7:** Test efficiency (s) of different approaches on remote sensing images.

Dimensions	25	50	75	100
SRC	4.1456	7.4306	8.1706	9.4669
AJRC	105.14	108.93	113.11	117.32
D-AJSR(1)	80.947	86.431	93.100	98.211
D-AJSR(4)	83.539	88.825	95.432	100.30
D-AJSR(8)	86.431	93.487	98.655	102.85
D-AJSR(16)	92.015	100.29	104.73	108.56
D-AJSR(32)	103.81	112.49	117.45	123.76
D-AJSR(64)	121.00	131.29	132.51	134.70

## Data Availability

The YaleB data sets and AR data sets are the public data sets, which can be found from reference [14, 15].
